# Characterization of Five Fungal Endophytes Producing Cajaninstilbene Acid Isolated from Pigeon Pea [*Cajanus cajan* (L.) Millsp.]

**DOI:** 10.1371/journal.pone.0027589

**Published:** 2011-11-15

**Authors:** Yuan Gao, Jin Tong Zhao, Yuan Gang Zu, Yu Jie Fu, Wei Wang, Meng Luo, Thomas Efferth

**Affiliations:** 1 Key Laboratory of Forest Plant Ecology, Ministry of Education, Northeast Forestry University, Harbin, People's Republic of China; 2 Engineering Research Center of Forest Bio-Preparation, Ministry of Education, Northeast Forestry University, Harbin, People's Republic of China; 3 Department of Pharmaceutical Biology, Institute of Pharmacy and Biochemistry, University of Mainz, Mainz, Germany; Nanjing Agricultural University, China

## Abstract

Five fungal endophytes (K4, K5, K6, K9, K14) producing Cajaninstilbene acid (CSA, 3-hydroxy-4-prenyl-5-methoxystilbene-2-carboxylic acid) were isolated from the roots of pigeon pea [*Cajanus cajan* (L.) Millsp.]. CSA is responsible for the prominent pharmacological activities in pigeon pea. The amount of CSA in culture solution varied among the five fungal endophytes. K4 produced the highest levels of CSA (1037.13 µg/L) among the endophytes tested after incubation for five days. Both morphological characteristics and molecular methods were used for species identification of fungal endophytes. The five endophytic isolates were characterized by analyzing the internal transcribed spacer (ITS) rRNA and β-tubulin genes. The K4, K5, K9 and K14 strains isolated from pigeon pea roots were found to be closely related to the species *Fusarium oxysporum*. K6 was identified as *Neonectria macrodidym*. The present study is the first report on the isolation and identification of fungal endophytes producing CSA in pigeon pea. The study also provides a scientific base for large scale production of CSA.

## Introduction

Pigeon pea [*Cajanus cajan* (L.) Millsp.], also called red gram, Congo pea, Gungo pea and no-eye pea, is a member of the legume family (*Leguminosae*). It is mainly distributed in tropical and subtropical countries as a substitute for expensive animal protein sources [1]. Pigeon pea has also been widely used as a traditional folk medicinal plant. The leaves of pigeon pea have been reported to arrest blood flow, relieve pain and kill worms [2]. They can be used to treat hepatitis, measles, dysentery, jaundice, diarrhea, sores, cough, bronchitis, bladder-stones, diabetes and many other illnesses [3]–[7].

In our previous work, the extracts of pigeon pea leaves showed significant antioxidant, antibacterial and antiherpetic activities [8]–[10]. The extracts also have hypoglycemic activity and potential in the treatment of postmenopausal osteoporosis [11]. Pigeon pea leaves are rich in flavonoids and stilbenes, which are known for their beneficial influence on human health [11]–[13].

**Figure 1:**
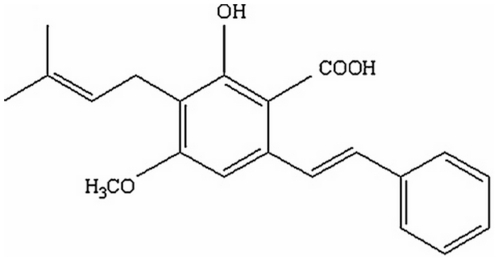
Chemical structure of cajaninstilbene acid.

Cajaninstilbene acid (CSA), 3-hydroxy-4-prenyl-5-methoxystilbene-2-carboxylic acid, is the main active constituent in pigeon pea leaves and exists in few plants [14] other than pigeon pea. The chemical structure of CSA is shown in Fig. 1. Though the general biosynthetic pathway of CSA in pigeon pea has not been elucidated in detail, the biosynthetic pathway of plant stilbenes has been well studied [15]. A large body of evidence shows that stilbenes participate in both constitutive and inducible defense mechanisms in plants [15]. Certain stimuli such as UV light, NO, H_2_O_2_, jasmonic acid and fungal infections induce the accumulation of stilbenes.

We hypothesized that endophytes in pigeon pea may contribute to CSA production. Endophytes infect living plants tissues without causing apparent disease symptoms in the host plant [16]. Endophytes are ubiquitous in a wide variety of plant species and most have a mutualistic relationship with their host [17]. Endophytes can improve plant adaptation to stress, increase biomass, or promote resistance to pathogen damage [18]–[20]. Some endophytes are thought to prevent their host from attacks by fungi, pests and mammals by producing secondary metabolites. Currently, endophytes are viewed as an outstanding source of bioactive products [21], [22]. Fungal endophytes possess a broad synthetic capability and synthesize abundant secondary metabolites with potential economic significance. Some endophytes produce biologically active constituents that are similar to or the same as those produced by their host, such as one fungal endophyte that produces taxol [23]. In addition, the production of secondary metabolites with pharmaceutical relevance is relatively common in endophytes, and thus they have great potential value as a source of drugs [24], [25]. Therefore, investigating endophytes represents a promising strategy to identify novel nature compounds. Endophytes can be cultured easily and quickly, and biomass can be accumulated by large scale fermentation, which is a unique advantage compared to plant resources [26].

The fungus *Fusarium* sp. has been isolated from a wide variety of sources, including soil, crops, plants and animals [27]. There are many non-pathogenic strains of *Fusarium* sp. including endophytes that utilize unique metabolic pathways to protect their host plants from invasion by pathogenic strains, pests and mammals [28]. Identification of *Fusarium* species has always been difficult due to ill-defined phenotypic classification systems [29], [30]. *Fusarium* taxonomy is primarily based on morphological characteristics of the anamorph, including the size and shape of macroconidia, the presence or absence of microconidia and chlamydospores, colony colour, and conidiophore structure [29], [30]. These characteristics can be highly variable depending on media and culturing conditions [31]. In addition, degeneration of cultures and growth of mutants may further complicate fungal identification [32]–[35]. Most teleomorphs of *Cylindrocarpon* sp. are currently classified as *Neonectria* [36], [37]. *Neonectria* sp. has also been reported to exhibit strong antimicrobial activity [38]. *Neonectria* taxonomy is based on anatomical characters of the perithecial wall and partly on characters of ascospores [39], [40].

A number of molecular tools have been developed to support morphological identifications. One of the most powerful techniques used in fungal identification is nucleotide sequencing [41]. Analysis of 18S ribosomal RNA sequences, usually used in phylogenetic analysis at higher taxonomic levels, is generally not adequate to differentiate fungal species. However, the internal transcribed spacer (ITS) regions ITS1 and ITS2, flanking the 5.8S rRNA gene is very useful in distinguishing species in many eukaryotic organisms including fungi [42]. Nuclear protein-coding loci, namely β-tubulin, are also highly substituted and can be used for identification of fungi [43]. The close relationship between *Fusarium* species is supported by the ITS and β-tubulin sequences [44]. The accurate identification of five fungal endophytes should be correlated by both the description of morphological characters and phylogenetic analysis of ITS and β-tubulin gene sequences.

Although many fungal endophytes isolated from healthy plants have been described, there are no reports on fungal endophytes in pigeon pea. Pigeon pea endophytes are worth investigating because of their potential to produce CSA or other valuable bioactive compounds.

In this manuscript, fungal endophytes were isolated from pigeon pea and screened for CSA production. Endophytes producing CSA may provide a new valuable medicinal resource in addition to plant resources. Five CSA-producing fungal isolates were then identified based on morphological and molecular parameters, in particular phylogenetic relatedness in two genes: ITS and β-tubulin. The present study may also provide a basis for the study of CSA biosynthesis.

## Results

### 1. Isolation of endophytes

**Figure 2:**
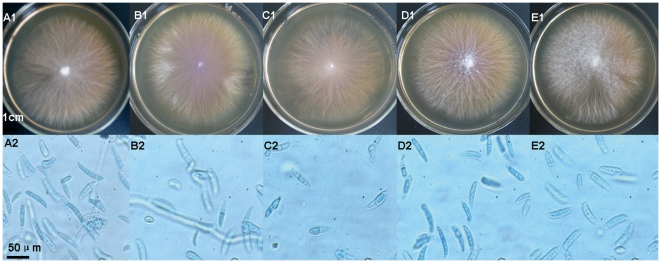
Colonies on PDA plates and photomicrographs of endophytes. A–E represent endophytes K4, K5, K6, K9 and K14. A1 to E1 show K4, K5, K6, K9 and K14 colonies on PDA medium after incubation for 14 days; A2 to E2 show photomicrographs of K4, K5, K6, K9 and K14 spores.

Endophytes were isolated from leaves, stems and roots of pigeon pea plants (72.2%, 76.2% and 67.4% of samples respectively). All endophytes grew 0.2–0.5 cm per day on PDA agar plate at 28°C. In the first 7 days, most fungal colonies on PDA were white and difficult to distinguish. After culturing for several more 7 days, different isolates were colored red, brown or black, and soft or rigid domes gradually formed in the center of colonies. Five CSA-producing isolates with similar biological characteristics were simultaneously found in roots of pigeon pea (Fig. 2). Fig. 2 shows the morphological characters of the CSA-producing endophytes K4, K5, K6, K9 and K14 on PDA agar plates. Fig. S1 shows the intercellular colonization of endphytes in the roots of pigeon pea.

K4 and K5 isolates produced flat-growing colonies up to 7.0 cm diam. in two weeks. The colonies consisted of white aerial mycelium tinged with purple, which progressed to deep purple with aging and released purple pigment and droplets into the PDA. Spores were elliptical in shape, conidia were (30.0–60.8) µm×(7.8–12.0) µm. K9 and K14 isolates grew in flat colonies up to 6.5 cm diam. in two weeks. The colonies were white but turned pink with aging and released pink pigment into the PDA. Spore shape was falcate or elliptical and conidia were (40.3–80.4) µm×(8.0–13.6) µm. The K6 isolate produced flat-growing colonies up to 4.5 cm diam. in two weeks. Colonies were white with yellow in the center. Spore shape was cylindrical and conidia were (23.2–49.2) µm×(6.0–12.8) µm. Colonies and spore morphology of all isolates revealed them to be closest to strains of *Fusarium* sp. and *Neonectria* sp.

### 2. Quantitative determination of CSA

**Figure 3:**
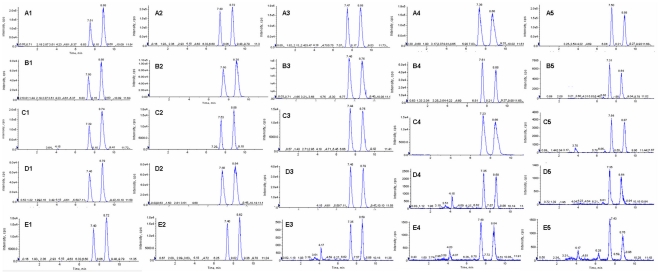
Representative LC–MS/MS chromatograms of endophyte fermentation extracts of K4, K5, K6, K9 and K14. A1–A5 show representative LC–MS/MS chromatograms of K4 after 5, 10, 15, 20 and 25 days of incubation; B1–B5 show representative LC–MS/MS chromatograms of K5 after 5, 10, 15, 20 and 25 days of incubation; C1–C5 show analogous representative LC–MS/MS chromatograms of K6; D1–D5 show analogous representative LC–MS/MS chromatograms of K9; E1–E5 showed representative LC–MS/MS chromatograms of K14.

**Figure 4:**
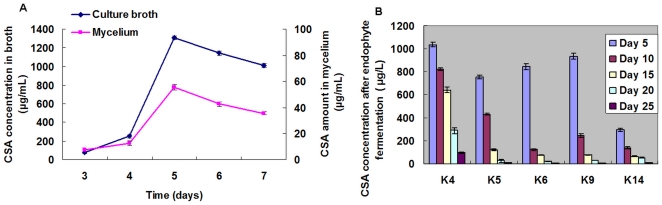
CSA concentration in mycelium and culture broth of endophyte K4 (A) (day 3–7) and in culture broth of five endophyte strains (day 5–25) (B). The fungal Mycelium extracts treating by 80% ethanol and culture liquid were extracted by ethyl acetate three times. The evaporated ethyl acetate extracts were subsequently dissolved in chromatogram methanol for determination of CSA production by LC-MS/MS.

LC-MS/MS analysis showed that five of the pigeon pea endophytes tested produced CSA. The endophyte extracts shared the same LC-MS/MS retention time for CSA as the CSA standard (Fig. 3). Most of the endophytes producing a substantial amount of CSA were closely related to *Fusarium* sp. and *Neonectria* sp. CSA concentration in broth and mycelium of K4 endophyte is presented in Fig. 4A. CSA concentrations in broth and mycelium show a similar trend. The concentration of CSA increased from 3 to 5 days (77.91 µg/L to 1037.13 µg/L) and then declined. It is indicated that CSA is synthesized rapidly by endophyte's first growth stage in cultures, the accumulation of CSA in culture are both in total and relative to mycelial mass. Among the CSA-producing endophytes, the concentration of CSA varied with culture time. The K4 endophyte has the highest CSA concentration (1037.13 µg/L) after incubation for 5 days and the lowest concentration (97.33 µg/L) after 25 days. K5 showed a similar trend to K4. The highest CSA concentration (755.00 µg/L) was found after incubation 5 days and the lowest concentration (9.45 µg/L) after 25 days. The K6, K9 and K14 endophytes revealed a similar trend (Fig. 4A). Under the same culturing conditions used to measure CSA production, a compound with the same molecular weight and ion fragments as CSA appeared at 7.50 minutes. This compound's concentration gradually increased with incubation time, and it may be an isomer of CSA (Fig. 3).

### 3. Molecular identification of endophytes

**Figure 5:**
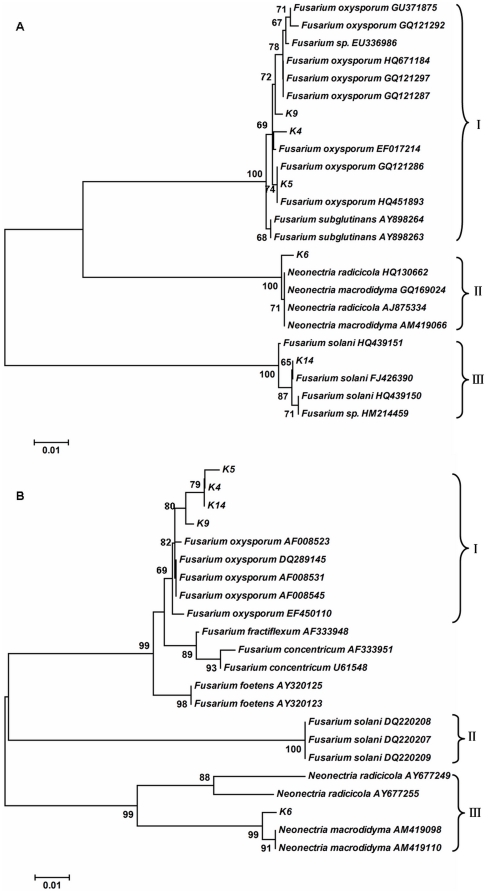
Phylogenetic reconstruction based on two gene sequences of endohytes. ITS1–5.8S–ITS2 sequences (A) and β-tubulin gene sequences (B) of endophytes were used for phylogenetic reconstruction. The trees were constructed using a neighbour-joining distance matrix. Evolutionary distances were computed using the Maximum Composite Likelihood method. Bootstrap values (1000 tree interactions) are indicated at the nodes.

**Figure 6:**
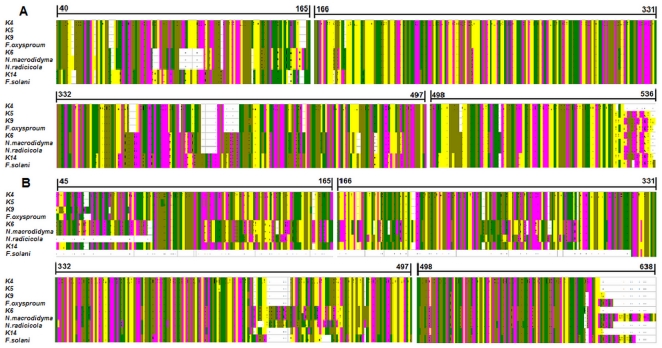
Alignment of ITS and β-tubulin gene sequences from five endophytes with reference sequences. (A) The master alignment of ITS sequences from K4 to K14 with *F. oxysporum*, *F. solani*, *N. radicicola* and *N. macrodidym*. The position of the first and final nucleotide alignment are highlighted. (B) The master alignment of β-tubulin gene sequences from K4 to K14 with *F. oxysporum*, *F. solani*, *N. radicicola*, *N. macrodidym*. The position of the first and final nucleotide alignment are highlighted. Nucleotides are shown by color bars (A: yellow, G: purple, T: green, C: brown).

Neighbour-joining (NJ) trees inferred from the aligned ITS1–5.8S–ITS2 and β-tubulin genes suggested that the K4, K5, K9 and K14 isolates were most closely related to *Fusarium oxysporum* (Fig. 5). In the ITS1–5.8S–ITS2 sequences, K4, K9 and K14 were different from *F. oxysporum* at one out of 495, four out of 501 and two out of 518 base positions, respectively. K5 was a 100% match with *F. oxysporum*. In β-tubulin genes sequences, K4, K5, K9 and K14 were different from *F. oxysporum* at two out of 526, five out of 526, one out of 526 and one out of 521 base positions, respectively. ITS1–5.8S–ITS2 and β-tubulin gene sequences of K6 were most similar to those of *N. radicicola* and *N. macrodidyma* (Fig. 6), respectively. Alignment of the ITS1–5.8S–ITS2 sequences of K6 indicated that K6 was different from *N. radicicola* at two out of 499 base positions and from *N. macrodidyma* at four out of 498 base positions. In β-tubulin gene sequences, K6 was different from *N. macrodidyma* at two out of 554 base positions and from *N. radicicola* at 66 of 505 base positions.

Morphological and biological characteristics of the endophytes also supported the conclusions reached through sequencing. In summary, the alignment of sequences of the five endophytes tested with known related species was used to distinguish differences between the two (Fig. 6).

## Discussion

By means of a highly sensitive LC-MS/MS based method, the present study verified that several fungal endophytes isolated from pigeon pea produce CSA. CSA production is higher in the leaves than in the roots of pigeon pea [3]. These results are based on the assumption that CSA may be synthesized in both roots and leaves. CSA is unstable and is transferred to other structures within the roots under the alkaline conditions created by the nitrogen fixation of leguminous pigeon pea roots. However, it can be detected in the leaves even in an acidic environment. However, the culturing medium used provides a stable pH that is suitable for the endophytes' CSA production. By this method, CSA can be detected in root endophytes. The isolation of endophytes from leaves is still in progress. Preliminary evidence suggests leaf endophytes can produce a limited amount of CSA (unpublished data).

Among the endophytes isolated from roots, the CSA generation rate varied with culture time. The results (Fig. 4A) confirmed that CSA was biosynthesized by the endophyte and biosynthesis initially proceeded quickly. The production of CSA occurs only during rapid mycelial growth as after incubate 5 days, the total concentration in the medium and the concentration relative to mycelial mass declined at a similar rate. Fig. 4B demonstrated differences between different strains in the rate of decline from maximum accumulation 5 to 25 days. Among these endophytes, K4 showed the maximum accumulation and slow decline rate. Meanwhile, an isomer of CSA was also detected and its amount gradually increased during the incubation time. Hence, we suppose that CSA maybe converted into this isomer during incubation.

Certain stimuli such as UV light, NO, H_2_O_2_, jasmonic acid and fungal infections induce the accumulation of stilbenes [15], all environment factors maybe contribute to the CSA biosynthesis. Meanwhile, the genes for CSA biosynthesis may be transferred between endophytes and plant cells by horizontal gene transfer, leading to the production of CSA by endophyte itself. As a result of these long-held associations, it is plausible that some of the endophytes may have developed genetic systems promoting the transfer of information between themselves and the host plant [24]. Further studies are warranted to test this hypothesis. The present study may facilitate the elucidation of the biosynthetic pathway for CSA. The presence of CSA-producing endophytes in pigeon pea supports the hypothesis that during the long co-evolution of endophytes and their host plants, one way in which endophytes adapted to their particular microenvironments was through the uptake of host plant DNA into their own genomes. This could have led to endophytes with the ability to synthesize phytochemicals originally associated with the host plant [45], [46].

*Fusarium* sp. and *Neonectria* sp. are very difficult to distinguish by their morphological characteristics. The close relationship between these species is supported by the ITS and β-tubulin sequences [44].

The morphology of the five isolated endophytes suggested a close relationship with *Fusarium* sp. and *Neonectria* sp.. Phylogenetic analysis of ITS and β-tubulin sequences supported the identification of the K4, K5, K9 and K14 as *F. oxysporum*. Identification of K6 was not as simple as that of K4, K5, K9 and K14, since analyzing different sequences provided inconclusive results. As the ITS region is generally considered to be less conserved than either small or large subunits of rRNA genes [47], the ITS analysis may be more informative in distinguishing closely related species. A single two base substitutions out of 499 positions in ITS1–5.8S–ITS2 between K6 and *Neonectria radicicola* compared to four base substitutions out of 498 positions for K6 and *N. macrodidyma* suggests that K6 may be more closely related to *N. radicicola*. However, in the β-tubulin gene sequences, K6 differs from *N. macrodidyma* at two out of 554 base positions and from *N. radicicola* at 66 out of 505 base positions, indicating that K6 is more different form *N. radicicola*. Considering both sequences, we conclude that K6 is more closely related to *N. macrodidyma*.

In the ITS1–5.8S–ITS2 tree generated with neighbour-joining analyses using the Maximum Composite Likelihood method, isolate K6 formed a cluster clade II supported by 100% bootstrap value with *N. radicicola* and *N. macrodidyma* and showed 99% similarity with both in BLAST analysis. Clade I composed of the isolates K4, K5 and K9 showed 99%, 100% and 99% similarity to *F. oxysporum* in BLAST analysis, and the bootstrap of clade I was 100%. Isolate K14 showed 99% similarity to *F. solani* in BLAST analysis, but the bootstrap analysis did not support the conclusion well (<70%).

According to the Neighbor-Joining (NJ) tree based on the β-tubulin gene sequences, isolates of K6 form a well-defined clade III with *N. macrodidyma* (bootstrap support value of 99%). Isolates in the “*F. oxysporum*” complex clade I (82% bootstrap support) are mainly in basal positions with a small number of defined clades with high bootstrap support values. K4, K5 and K14 both showed 99%, similarity to *F. oxysporum* in BLAST analysis, and the bootstrap value was 79%. K9 showed 99% similarity to *F. oxysporum* in BLAST analysis, and the bootstrap value was 80%.

Firstly, our results indicate that the endophytes produce CSA. Endophytes producing valuable products could not only reduce the use of slow-growing and rare plants in industrial production but also preserve the world's ever-diminishing biodiversity. Furthermore, using a microbial source to produce a certain valued product such as CSA may be easier and more economical, effectively reducing the product's market price [48]. Endophytes can be easily cultured in vitro and accumulate biomass through large-scale fermentation in a process distinctive from that for plants [26]. In the future, CSA may be more quickly and efficiently obtained from endophytes than from plants. Therefore, CSA-producing endophytes from pigeon pea may provide valuable natural medicinal resources for large-scale production of CSA. Further optimization of submerged cultivation conditions and molecular studies of K4 are merit future investigation.

## Materials and Methods

### 1. Isolation of fungal endophytes

Fungal endophytes were isolated from healthy roots, stems and leaves of pigeon pea [*Cajanus cajan* (L.) Millsp.]. Fresh tissue of pigeon pea were collected from the arboretum of the Key Laboratory of Forest Plant Ecology, Ministry of Education, Northeast Forestry University, Harbin, PR China, Voucher specimens (No. 052056001001001) were deposited in the herbarium of this Key Laboratory. The plant materials were subjected to endophyte isolation within 3 hours of harvest. Specifically, the roots, stems and leaves of pigeon pea were washed with running tap water, sterilized with 75% ethanol for 60, 60 and 30 seconds, rinsed in sterile water and 5% sodium hypochlorite for 7, 5, and 3 minutes, rinsed in sterile water three times respectively, and finally cut into 1 cm long segments. Both edges of sterilized segments were cut off, and the segments were incubated at 28±1°C on potato dextrose agar (PDA) medium for 14–21 days until the colonies originating from the newly formed surface of the segments appeared. Penicillin (20 µg/mL) was added to culture medium to suppress bacterial growth. The effectiveness of the surface sterilization procedure was verified by plating uncut plant tissue and samples of the final rinse water. Furthermore, the purified fungal endophytes were numbered and transferred to fresh PDA slants separately, and these slants were incubated for 14–21 days and then kept at 4°C.

Isolates of five endophytes were identified according to their cultural and morphological characteristics as described by Nelson *et al.* (1983) [30]. The diameter and colour of colonies was measured and determined. The microscopic features such as size and shape of hyphae and conidia were examined and measured under a light microscope after maturation.

### 2. Preparation the endophytes samples and standard solutions

For quantitative determination of CSA, 15 conical flasks of 500 mL capacity were used, with each endophyte isolate cultured in three parallel samples containing 200 mL Potato Dextrose Broth (PDB) medium. Fungal endophytes were grown in PDB medium and cultured for 5, 10, 15, 20 and 25 days at 28°C with shaking (180 rev/min) on a rotary shaker. The fungal culture liquid was then filtered and then endophytes extracted by ethyl acetate three times. Mycelium was rinsed in sterile water three times, oven for 48 h at 40°C, then ultrasonic processing 30 min in 80% ethanol and extracted by ethyl acetate three times. The evaporated ethyl acetate extracts were subsequently dissolved in chromatogram methanol and filtered with a 0.22 µm member filter, before being used for determination of CSA production.

A stock solution of reference sample CSA was prepared in methanol at a final concentration of 100 µg/mL. The final concentrations of the standard curve samples were 10, 20, 50, 100, 200 500, 1000, 3000 and 6000 ng/mL. The standard stock solution and working standard solutions were all stored at 4°C. Standard calibration samples were stored at −20°C until analysis.

### 3. Quantitative determination of CSA by LC-MS/MS

The determination of CSA based on LC-MS/MS methods described by Hua *et al.* (2010) [49]. An Agilent 1100 series HPLC system (Agilent Technologies, San Jose, CA, USA) consisting of a G1312A binary pump, a 7725i manual injector, and a G1379A degasser were used for chromatographic analysis. Chromatographic separations were achieved on a HIQ Sil C18 column (4.6 mm×250 mm, KYA TEACH, made in Japan) maintained at 20°C. The column effluent was monitored by an API3000 triple-stage quadrupole mass spectrometer (Applied Biosystems, Concord, Canada) equipped with an electrospray ionization (ESI) source. The mobile phase consisted of water and methanol (9∶91, v/v) containing 0.1% formic acid. The flow rate was 1.0 mL/min, and the sample injection volume was 10 µL.

The ion spray voltage was set at −4500 V. Compound parameters viz., declustering potential (DP), collision energy (CE), entrance potential (EP) and collision exit potential (CXP) were set at −60, −25, −10, −5 V respectively for analysis of CSA. The mass spectrometer was operated in ESI negative ion mode, and the detection of the ions was performed in the multiple reaction monitoring (MRM) mode, monitoring the transition of m/z 337.1 precursor ion [M–H]^−^ to the m/z 293.0 product ion for CSA. The compound of second peak were detected by LC-MS/MS in the same conditions as used to monitor the transition of m/z 337.1 precursor ion [M–H]^−^ to the m/z 293.0 product ion.

### 4. Fungal genomic DNA extraction

The fungal genomic DNA extraction procedure was followed by extraction using a universal genomic DNA extraction kit (Tiangen, Beijing, China). After incubation for two weeks on PDA, fungal mycelia was harvested, frozen in liquid nitrogen, and ground to powder with a mortar. Subsequently, fungal genomic DNA was extracted and purified following the manufacturer's instructions. The genomic DNA was determined by electrophoresis through 1% agarose gel stained with ethidium bromide. The concentration of DNA was determined by an RNA/DNA Calculator Gene Quant II (Pharmarcia Biotech Amersham, UK).

### 5. ITS and β-tublin amplification and sequencing

The ITS region of the ribosomal DNA was amplified using the primers ITS1 (5′-TCCGTAGGTGAACCTGCGG-3′) and ITS4 (5′-TCCTCCGCTTATTGATATG-C-3′) [50]. The β-tubulin gene was amplified using the primers T1 (5′-ATGCGTGAGATTGTAAGT-3′) and T22 (5′-TGACCGAAAACGAAGTTGTC-3′) [51]. The PCR reaction mixtures used to amplify the fragments consisted of final concentrations of 1×PCR buffer and contained Pfu DNA Polymerase (Sangon, Shanghai, China), 0.5 µM forward and reverse primers, and approximately 30 ng of fungal genomic DNA made up to a total reaction volume of 50 µL with sterile deionised water. Amplification was performed in an ST1000™ thermo cycler (BioRad, California, USA). The amplification program was as follows: 1 cycle of 3 minutes at 95°C, 30 cycles of 30 seconds at 95°C, 30 seconds at 55°C, 1 minute at 72°C, and finally 1 period of 5 minutes at 72°C. Amplified fragments were separated by electrophoresis through 1% agarose gels stained with ethidium bromide and purified using Gel Product Purification Kit (Tiangen, Beijing, China). DNA fragments were sequenced by the BigDye Terminator v3.1 Cycle Sequencing kit (Applied Biosystems) and an ABI 3730XL sequencer (Applied Biosystems) at the Sangon Biotech Co., Ltd (Shanghai, China).

### 6. Analysis of DNA sequences

ITS1–5.8S–ITS2 and β-tubulin gene sequences were successfully amplified from each CSA-producing endophyte by means of PCR. The obtained sequences were screened for homology using the MEGA version 4.1 [52] (http://www.ncbi.nlm.nih.gov/BLAST). Sequence data has been submitted to Genbank under the accession numbers JF807393 to JF807397 and JF807403 to JF807407. When sequence identity was greater than 97% homologous, genus and species of the database results were accepted; when identity was 97 to 95% homologous, only genus was accepted [53]. To determine the taxonomic identity of the endophytes, the ITS sequences and β-tubulin gene sequences from both the CSA-producing endophytes and species thought to be of close homology (Table 1) within the *Fusarium* family were aligned using the Clustal of alignment in MEGA version 4.1 [52]. Phylogenetic analysis was then conducted with phylogeny in MEGA version 4.1. A neighbour-joining tree [54] was created from the alignment file using the Maximum Composite Likelihood method and bootstrapping of 1000 replicates [55].

**Table 1:** Sequences and GenBank accession numbers by DNA locus.

Fungal taxon	Source and authors	Accession No.
*Fusarium sp.*	Sun, H. and Song, R. 2007	EU338986 (ITS)
*Fusarium sp.*	Zhao, X. et al. 2010	HM214459 (ITS)
*Fusarium oxysporum*	Yang, X. M. 2009	GU371875 (ITS)
*Fusarium oxysporum*	Manici, L. M. and Caputo, F. 2009	EF017214 (ITS)
*Fusarium oxysporum*	Shanmugam, V. 2009	GQ121286 (ITS)
*Fusarium oxysporum*	Shanmugam, V. 2009	GQ121297 (ITS)
*Fusarium oxysporum*	Shanmugam, V. 2009	GQ121292 (ITS)
*Fusarium oxysporum*	Cheng, J. et al. 2010	HQ671184 (ITS)
*Fusarium oxysporum*	Lang, C. Y. et al. 2010	HQ451893 (ITS)
*Neonectria sp.*	Mitter, B. et al. 2010	HQ130662 (ITS)
*Neonectria radicicola*	Oliveira, H. and Nascimento, T. C. 2008	AJ875334 (ITS)
*Neonectria macrodidyma*	Liu, Y. et al. 2009	GQ169024 (ITS)
*Neonectria macrodidyma*	Oliveira, H. et al. 2006	AM419066 (ITS)
*Fusarium solani*	Sun, H. and Song, R. 2009	FJ426390 (ITS)
*Fusarium solani*	Lee, H. B. 2010	HQ439150 (ITS)
*Fusarium subglutinans*	Liu, X. and Wang, X. 2005	AY898264 (ITS)
*Fusarium subglutinans*	Liu, X. and Wang, X. 2005	AY898263 (ITS)
*Fusarium oxysporum*	Cigelnik, E. and O'Donnell, K.1998	AF008545 (β-tubulin)
*Fusarium oxysporum*	Cigelnik, E. and O'Donnell, K.1998	AF008531 (β-tubulin)
*Fusarium oxysporum*	Cigelnik, E. and O'Donnell, K.1998	AF008523 (β-tubulin)
*Fusarium oxysporum*	Cigelnik, E. and O'Donnell, K.1998	AF008522 (β-tubulin)
*Fusarium oxysporum*	Zhang, C. L. et al. 2006	DQ289145 (β-tubulin)
*Fusarium oxysporum*	Garcia-Sanchez. M. A. et al. 2007	EF450110 (β-tubulin)
*Neonectria macrodidyma*	Oliveira, H. et al. 2009	AM419098 (β-tubulin)
*Neonectria macrodidyma*	Oliveira, H. et al. 2009	AM419110 (β-tubulin)
*Neonectria radicicola*	Halleen, F. et al. 2004	AY677249 (β-tubulin)
*Neonectria radicicola*	Halleen, F. et al. 2004	AY677255 (β-tubulin)
*Fusarium foetens*	Schroers, H. J. et al. 2004	AY320123 (β-tubulin)
*Fusarium foetens*	O' Donnell, K. 2003	AY320125 (β-tubulin)
*Fusarium concentricum*	O' Donnell, K. et al. 1996	U61548 (β-tubulin)
*Fusarium concentricum*	O' Donnell, K. 2001	AF333951 (β-tubulin)
*Fusarium solani*	Bogale, M. et al. 2006	DQ220207 (β-tubulin)
*Fusarium solani*	Bogale, M. et al. 2006	DQ220208 (β-tubulin)
*Fusarium solani*	Bogale, M. et al. 2006	DQ220209 (β-tubulin)

## Supporting Information

Figure S1 **Intercellular colonization of endphytes (arrows) in the roots of pigeon pea.**
(DOC)
