# Supplementary figures and images for: Characterization of Five Fungal Endophytes Producing Cajaninstilbene Acid Isolated from Pigeon Pea [Cajanus cajan (L.) Millsp.]
Source: PLoS One. 2011 Nov 15;6(11):e27589. doi: 10.1371/journal.pone.0027589 (PMC3216958; doi:10.1371/journal.pone.0027589)

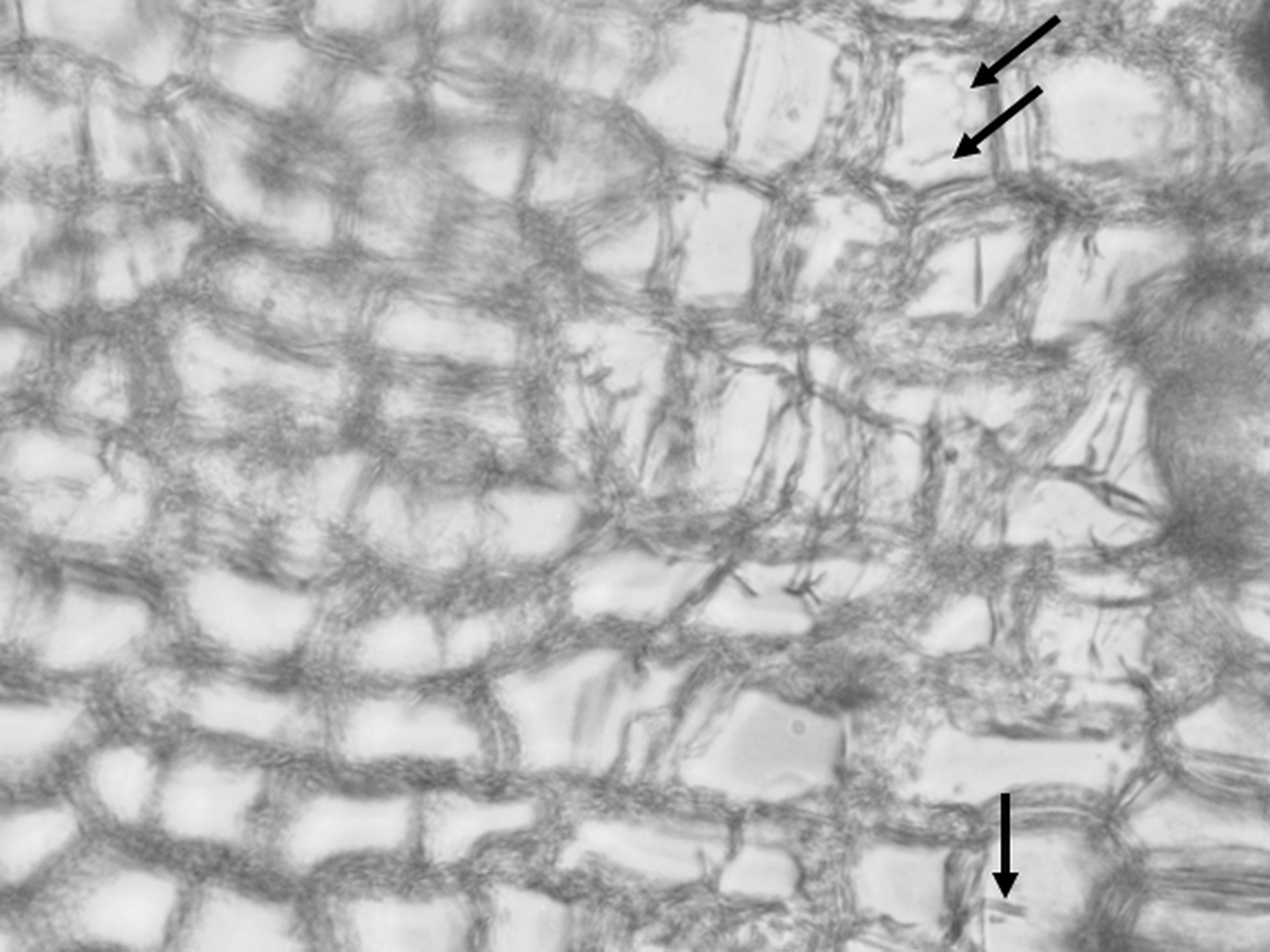


**Figure S1. Intercellular colonization of endphytes (arrows) in the roots of pigeon pea.**

Supplement: Figure S1 — Intercellular colonization of endphytes (arrows) in the roots of pigeon pea. (DOC) [file pone.0027589.s001.doc]
